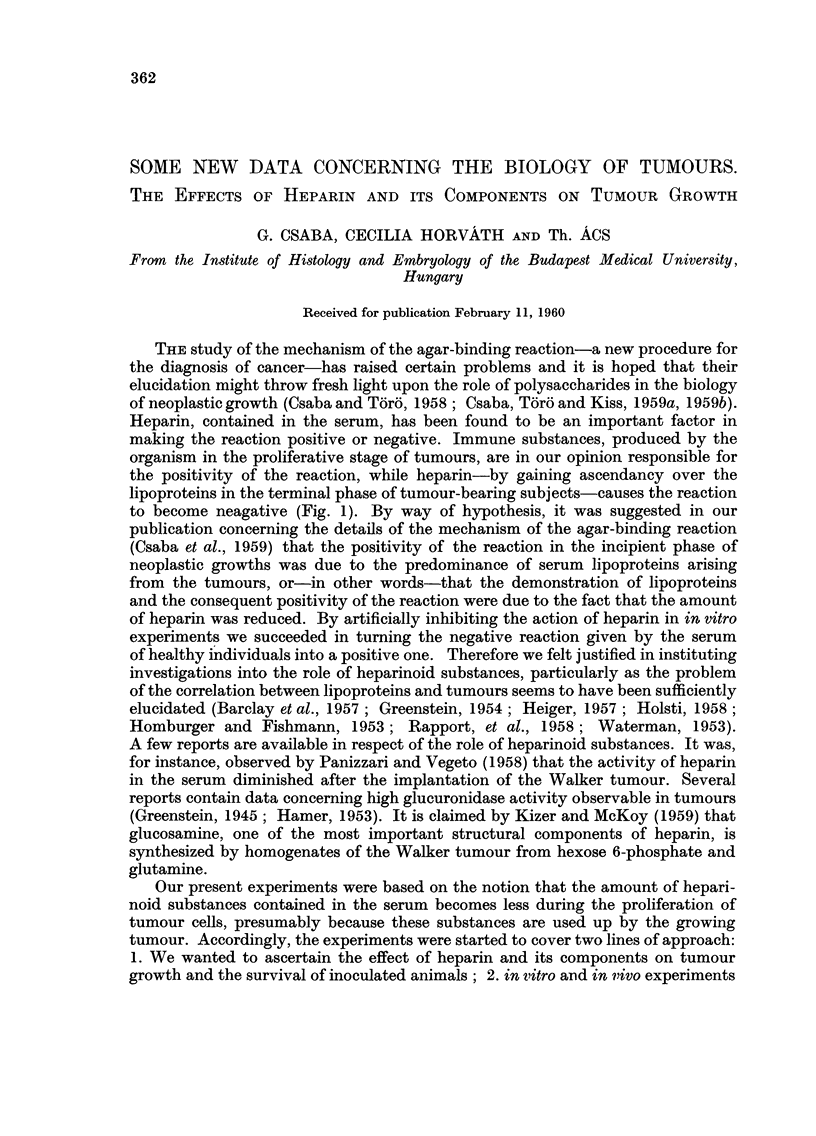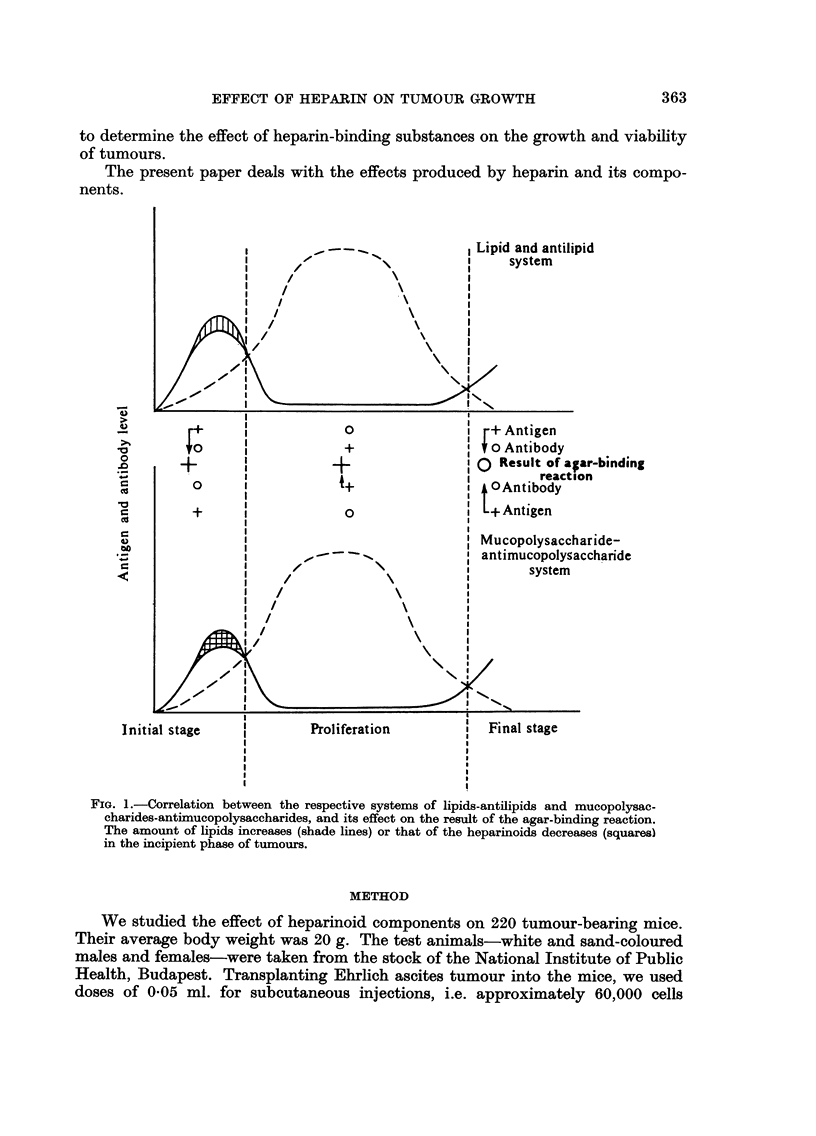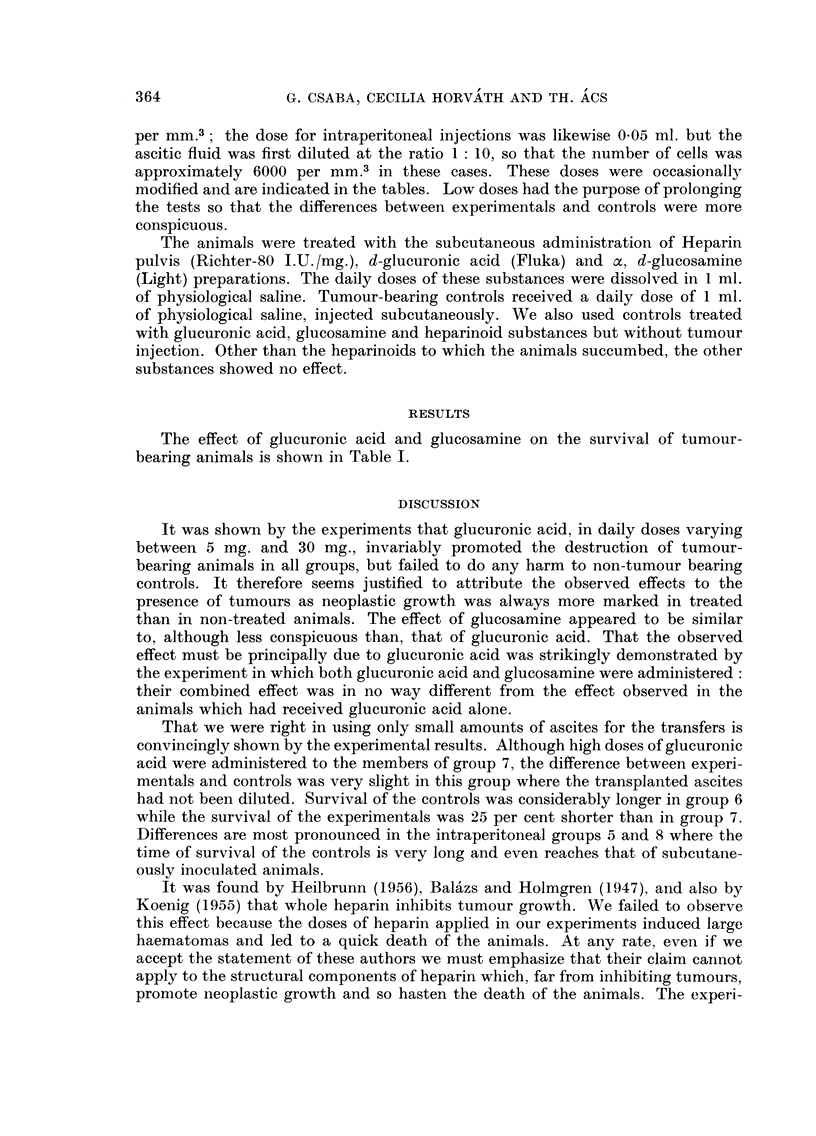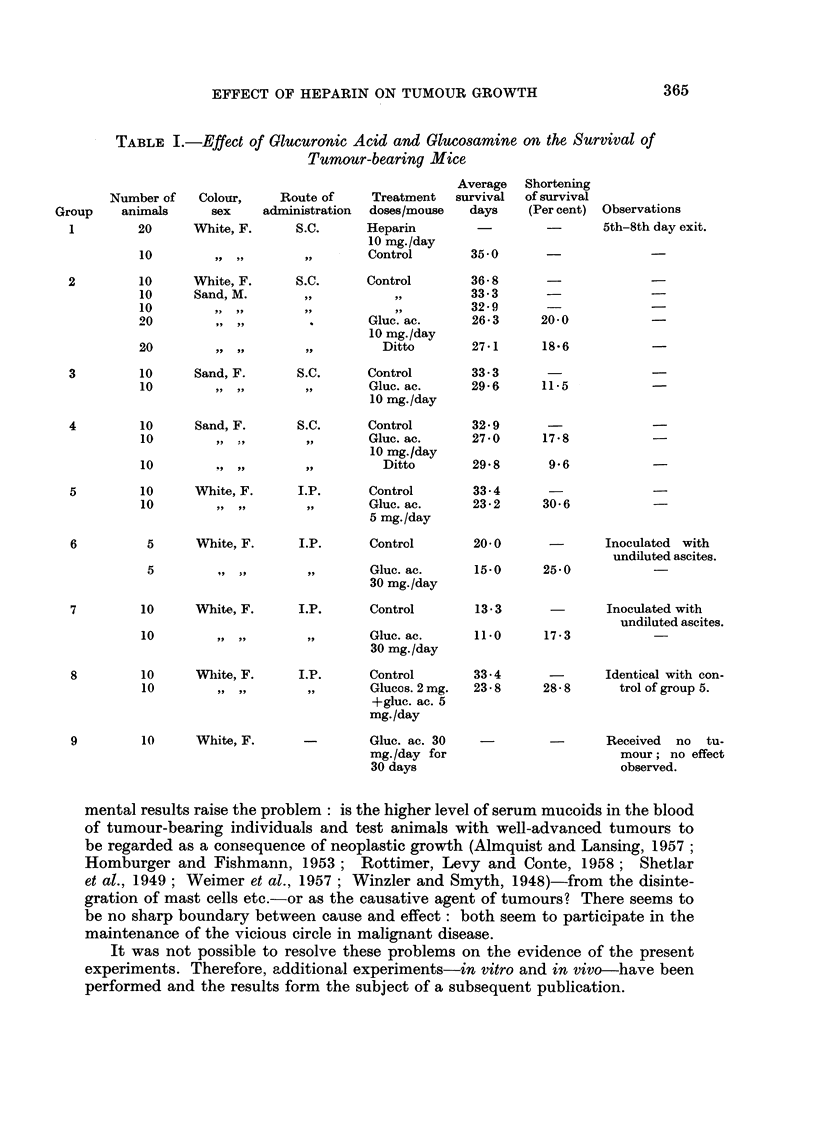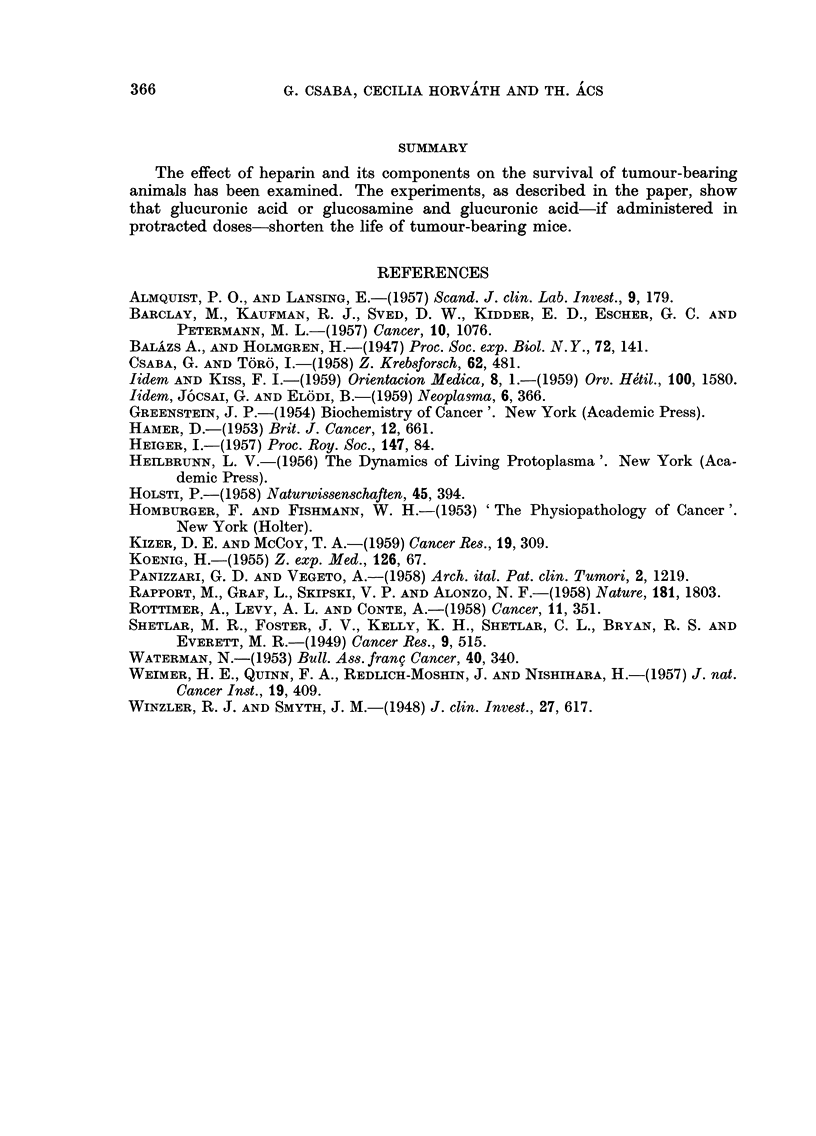# Some New Data concerning the Biology of Tumours

**DOI:** 10.1038/bjc.1960.42

**Published:** 1960-06

**Authors:** G. Csaba, Cecilia Horváth, Th. Ács


### SOME NEW DATA CONCERNING THE BIOLOGY OF TUMOURS.

### THE EFFECTS OF HEPARIN AND ITS COMPONENTS ON TUMOUR GROWTH

G. CSABA, CECILIA HORVÁTH AND Th. ÁCS

*From the Institute of Histology and Embryology of the Budapest Medical University, Hungary*

Received for publication February 11, 1960

THE study of the mechanism of the agar-binding reaction—a new procedure for the diagnosis of cancer—has raised certain problems and it is hoped that their elucidation might throw fresh light upon the role of polysaccharides in the biology of neoplastic growth (Csaba and Törö, 1958 ; Csaba, Törö and Kiss, 1959*a*, 1959*b*). Heparin, contained in the serum, has been found to be an important factor in making the reaction positive or negative. Immune substances, produced by the organism in the proliferative stage of tumours, are in our opinion responsible for the positivity of the reaction, while heparin—by gaining ascendancy over the lipoproteins in the terminal phase of tumour-bearing subjects—causes the reaction to become neagative (Fig. 1). By way of hypothesis, it was suggested in our publication concerning the details of the mechanism of the agar-binding reaction (Csaba *et al.*, 1959) that the positivity of the reaction in the incipient phase of neoplastic growths was due to the predominance of serum lipoproteins arising from the tumours, or—in other words—that the demonstration of lipoproteins and the consequent positivity of the reaction were due to the fact that the amount of heparin was reduced. By artificially inhibiting the action of heparin in *in vitro* experiments we succeeded in turning the negative reaction given by the serum of healthy individuals into a positive one. Therefore we felt justified in instituting investigations into the role of heparinoid substances, particularly as the problem of the correlation between lipoproteins and tumours seems to have been sufficiently elucidated (Barclay *et al.*, 1957 ; Greenstein, 1954 ; Heiger, 1957 ; Holsti, 1958 ; Homburger and Fishmann, 1953 ; Rapport, *et al.*, 1958 ; Waterman, 1953). A few reports are available in respect of the role of heparinoid substances. It was, for instance, observed by Panizzari and Vegeto (1958) that the activity of heparin in the serum diminished after the implantation of the Walker tumour. Several reports contain data concerning high glucuronidase activity observable in tumours (Greenstein, 1945 ; Hamer, 1953). It is claimed by Kizer and McKoy (1959) that glucosamine, one of the most important structural components of heparin, is synthesized by homogenates of the Walker tumour from hexose 6-phosphate and glutamine.

Our present experiments were based on the notion that the amount of heparinoid substances contained in the serum becomes less during the proliferation of tumour cells, presumably because these substances are used up by the growing tumour. Accordingly, the experiments were started to cover two lines of approach: 1. We wanted to ascertain the effect of heparin and its components on tumour growth and the survival of inoculated animals ; 2. *in vitro* and *in vivo* experiments to determine the effect of heparin-binding substances on the growth and viability of tumours.

The present paper deals with the effects produced by heparin and its components.

FIG. 1.—Correlation between the respective systems of lipids-antilipids and mucopolysaccharides-antimucopolysaccharides, and its effect on the result of the agar-binding reaction. The amount of lipids increases (shade lines) or that of the heparinoids decreases (squares) in the incipient phase of tumours.

### METHOD

We studied the effect of heparinoid components on 220 tumour-bearing mice. Their average body weight was 20 g. The test animals—white and sand-coloured males and females—were taken from the stock of the National Institute of Public Health, Budapest. Transplanting Ehrlich ascites tumour into the mice, we used doses of 0·05 ml. for subcutaneous injections, i.e. approximately 60,000 cells per mm.$^3$; the dose for intraperitoneal injections was likewise 0·05 ml. but the ascitic fluid was first diluted at the ratio 1 : 10, so that the number of cells was approximately 6000 per mm.$^3$ in these cases. These doses were occasionally modified and are indicated in the tables. Low doses had the purpose of prolonging the tests so that the differences between experimentals and controls were more conspicuous.

The animals were treated with the subcutaneous administration of Heparin pulvis (Richter-80 I.U./mg.), *d*-glucuronic acid (Fluka) and $\alpha$, *d*-glucosamine (Light) preparations. The daily doses of these substances were dissolved in 1 ml. of physiological saline. Tumour-bearing controls received a daily dose of 1 ml. of physiological saline, injected subcutaneously. We also used controls treated with glucuronic acid, glucosamine and heparinoid substances but without tumour injection. Other than the heparinoids to which the animals succumbed, the other substances showed no effect.

### RESULTS

The effect of glucuronic acid and glucosamine on the survival of tumour-bearing animals is shown in Table I.

### DISCUSSION

It was shown by the experiments that glucuronic acid, in daily doses varying between 5 mg. and 30 mg., invariably promoted the destruction of tumour-bearing animals in all groups, but failed to do any harm to non-tumour bearing controls. It therefore seems justified to attribute the observed effects to the presence of tumours as neoplastic growth was always more marked in treated than in non-treated animals. The effect of glucosamine appeared to be similar to, although less conspicuous than, that of glucuronic acid. That the observed effect must be principally due to glucuronic acid was strikingly demonstrated by the experiment in which both glucuronic acid and glucosamine were administered : their combined effect was in no way different from the effect observed in the animals which had received glucuronic acid alone.

That we were right in using only small amounts of ascites for the transfers is convincingly shown by the experimental results. Although high doses of glucuronic acid were administered to the members of group 7, the difference between experimentals and controls was very slight in this group where the transplanted ascites had not been diluted. Survival of the controls was considerably longer in group 6 while the survival of the experimentals was 25 per cent shorter than in group 7. Differences are most pronounced in the intraperitoneal groups 5 and 8 where the time of survival of the controls is very long and even reaches that of subcutaneously inoculated animals.

It was found by Heilbrunn (1956), Balázs and Holmgren (1947), and also by Koenig (1955) that whole heparin inhibits tumour growth. We failed to observe this effect because the doses of heparin applied in our experiments induced large haematomas and led to a quick death of the animals. At any rate, even if we accept the statement of these authors we must emphasize that their claim cannot apply to the structural components of heparin which, far from inhibiting tumours, promote neoplastic growth and so hasten the death of the animals. The experi-

TABLE I.—*Effect of Glucuronic Acid and Glucosamine on the Survival of Tumour-bearing Mice*

| Group | Number of animals | Colour, sex | Route of administration | Treatment doses/mouse | Average survival days | Shortening of survival (Per cent) | Observations |
|---|---|---|---|---|---|---|---|
| 1 | 20 | White, F. | S.C. | Heparin 10 mg./day | — | — | 5th–8th day exit. |
| | 10 | „ „ | „ | Control | 35·0 | — | — |
| 2 | 10 | White, F. | S.C. | Control | 36·8 | — | — |
| | 10 | Sand, M. | „ | „ | 33·3 | — | — |
| | 10 | „ „ | „ | „ | 32·9 | — | — |
| | 20 | „ „ | . | Gluc. ac. 10 mg./day | 26·3 | 20·0 | — |
| | 20 | „ „ | „ | Ditto | 27·1 | 18·6 | — |
| 3 | 10 | Sand, F. | S.C. | Control | 33·3 | — | — |
| | 10 | „ „ | „ | Gluc. ac. 10 mg./day | 29·6 | 11·5 | — |
| 4 | 10 | Sand, F. | S.C. | Control | 32·9 | — | — |
| | 10 | „ „ | „ | Gluc. ac. 10 mg./day | 27·0 | 17·8 | — |
| | 10 | „ „ | „ | Ditto | 29·8 | 9·6 | — |
| 5 | 10 | White, F. | I.P. | Control | 33·4 | — | — |
| | 10 | „ „ | „ | Gluc. ac. 5 mg./day | 23·2 | 30·6 | — |
| 6 | 5 | White, F. | I.P. | Control | 20·0 | — | Inoculated with undiluted ascites. |
| | 5 | „ „ | „ | Gluc. ac. 30 mg./day | 15·0 | 25·0 | — |
| 7 | 10 | White, F. | I.P. | Control | 13·3 | — | Inoculated with undiluted ascites. |
| | 10 | „ „ | „ | Gluc. ac. 30 mg./day | 11·0 | 17·3 | — |
| 8 | 10 | White, F. | I.P. | Control | 33·4 | — | Identical with control of group 5. |
| | 10 | „ „ | „ | Glucos. 2 mg. +gluc. ac. 5 mg./day | 23·8 | 28·8 | |
| 9 | 10 | White, F. | — | Gluc. ac. 30 mg./day for 30 days | — | — | Received no tumour; no effect observed. |

mental results raise the problem : is the higher level of serum mucoids in the blood of tumour-bearing individuals and test animals with well-advanced tumours to be regarded as a consequence of neoplastic growth (Almquist and Lansing, 1957 ; Homburger and Fishmann, 1953 ; Rottimer, Levy and Conte, 1958 ; Shetlar *et al.*, 1949 ; Weimer *et al.*, 1957 ; Winzler and Smyth, 1948)—from the disintegration of mast cells etc.—or as the causative agent of tumours? There seems to be no sharp boundary between cause and effect : both seem to participate in the maintenance of the vicious circle in malignant disease.

It was not possible to resolve these problems on the evidence of the present experiments. Therefore, additional experiments—*in vitro* and *in vivo*—have been performed and the results form the subject of a subsequent publication.

SUMMARY

The effect of heparin and its components on the survival of tumour-bearing animals has been examined. The experiments, as described in the paper, show that glucuronic acid or glucosamine and glucuronic acid—if administered in protracted doses—shorten the life of tumour-bearing mice.

REFERENCES

ALMQUIST, P. O., AND LANSING, E.—(1957) *Scand. J. clin. Lab. Invest.*, **9**, 179.
BARCLAY, M., KAUFMAN, R. J., SVED, D. W., KIDDER, E. D., ESCHER, G. C. AND PETERMANN, M. L.—(1957) *Cancer*, **10**, 1076.
BALÁZS A., AND HOLMGREN, H.—(1947) *Proc. Soc. exp. Biol. N.Y.*, **72**, 141.
CSABA, G. AND TÖRÖ, I.—(1958) *Z. Krebsforsch*, **62**, 481.
*Iidem* AND KISS, F. I.—(1959) *Orientacion Medica*, **8**, 1.—(1959) *Orv. Hétil.*, **100**, 1580.
*Iidem*, JÓCSAI, G. AND ELÖDI, B.—(1959) *Neoplasma*, **6**, 366.
GREENSTEIN, J. P.—(1954) Biochemistry of Cancer '. New York (Academic Press).
HAMER, D.—(1953) *Brit. J. Cancer*, **12**, 661.
HEIGER, I.—(1957) *Proc. Roy. Soc.*, **147**, 84.
HEILBRUNN, L. V.—(1956) The Dynamics of Living Protoplasma '. New York (Academic Press).
HOLSTI, P.—(1958) *Naturwissenschaften*, **45**, 394.
HOMBURGER, F. AND FISHMANN, W. H.—(1953) ' The Physiopathology of Cancer '. New York (Holter).
KIZER, D. E. AND MCCOY, T. A.—(1959) *Cancer Res.*, **19**, 309.
KOENIG, H.—(1955) *Z. exp. Med.*, **126**, 67.
PANIZZARI, G. D. AND VEGETO, A.—(1958) *Arch. ital. Pat. clin. Tumori*, **2**, 1219.
RAPPORT, M., GRAF, L., SKIPSKI, V. P. AND ALONZO, N. F.—(1958) *Nature*, **181**, 1803.
ROTTIMER, A., LEVY, A. L. AND CONTE, A.—(1958) *Cancer*, **11**, 351.
SHETLAR, M. R., FOSTER, J. V., KELLY, K. H., SHETLAR, C. L., BRYAN, R. S. AND EVERETT, M. R.—(1949) *Cancer Res.*, **9**, 515.
WATERMAN, N.—(1953) *Bull. Ass. franç Cancer*, **40**, 340.
WEIMER, H. E., QUINN, F. A., REDLICH-MOSHIN, J. AND NISHIHARA, H.—(1957) *J. nat. Cancer Inst.*, **19**, 409.
WINZLER, R. J. AND SMYTH, J. M.—(1948) *J. clin. Invest.*, **27**, 617.